# Are Smartwatches a Suitable Tool to Monitor Noise Exposure for Public Health Awareness and Otoprotection?

**DOI:** 10.3389/fneur.2022.856219

**Published:** 2022-03-23

**Authors:** Tim Fischer, Stephan Schraivogel, Marco Caversaccio, Wilhelm Wimmer

**Affiliations:** ^1^Hearing Research Laboratory, ARTORG Center for Biomedical Engineering Research, University of Bern, Bern, Switzerland; ^2^Department of ENT—Head and Neck Surgery, Inselspital, Bern University Hospital, University of Bern, Bern, Switzerland

**Keywords:** *L*_*Aeq*_, noise exposure, noise dosimetry, wearables, ecological assessment, otoprotection, tinnitus, big data

## Abstract

**Introduction and Objectives:**

Noise-induced hearing loss (NIHL) and tinnitus are common problems that can be prevented with hearing protection measures. Sound level meters and noise dosimeters enable to monitor and identify health-threatening occupational or recreational noise, but are limited in their daily application because they are usually difficult to operate, bulky, and expensive. Smartwatches, which are becoming increasingly available and popular, could be a valuable alternative to professional systems. Therefore, the aim of this study was to evaluate the applicability of smartwatches for accurate environmental noise monitoring.

**Methods:**

The A-weighted equivalent continuous sound pressure level (*L*_*Aeq*_) was recorded and compared between a professional sound level meter and a popular smartwatch. Noise exposure was assessed in 13 occupational and recreational settings, covering a large range of sound pressure levels between 35 and 110 dBA. To assess measurement agreement, a Bland-Altman plot, linear regression, the intra-class correlation coefficient, and descriptive statistics were used.

**Results:**

Overall, the smartwatch underestimated the sound level meter measurements by 0.5 dBA (95% confidence interval [0.2, 0.8]). The intra-class correlation coefficient showed excellent agreement between the two devices (ICC = 0.99), ranging from 0.65 (music club) to 0.99 (concert) across settings. The smartwatch's sampling rate decreased significantly with lower sound pressure levels, which could have introduced measurement inaccuracies in dynamic acoustic environments.

**Conclusions:**

The assessment of ambient noise with the tested smartwatch is sufficiently accurate and reliable to improve awareness of hazardous noise levels in the personal environment and to conduct exploratory clinical research. For professional and legally binding measurements, we recommend specialized sound level meters or noise dosimeters. In the future, smartwatches will play an important role in monitoring personal noise exposure and will provide a widely available and cost-effective measure for otoprotection.

## 1. Introduction

Intentionally or unintentionally, repeated exposure to loud noise irreversibly damages hearing by causing cell death to the inner ear's hair cells (1). A recent meta-analysis estimates the pooled prevalence of hazardous occupational exposure to noise among the general population of workers at 17% (2). The World Health Organization (WHO) guesses that outside the workplace, exposure to noise in recreational or social settings puts ~1.1 billion young people worldwide at risk of developing noise-induced hearing loss (NIHL) (3). In addition to NIHL, exposure to loud noise increases the risk of cardiovascular disease, hypertension, sleep disturbance, occupational accidents, tinnitus and reduced cognitive performance (4–8).

In acoustic environments with uncomfortably high noise levels, a natural reaction is to move away from the noise source or to use hearing protection [e.g., earplugs, earmuffs, or active noise cancellation; (9)]. However, it is not only the sound level, but its combination with the duration of exposure that causes NIHL (10). Therefore, protection concepts for noise commonly apply the so-called dose principle, in which the acoustic exposure is energetically averaged over a defined period of time. Many everyday situations exceed the tolerable acoustic dose (3, 11, 12). Because damage usually occurs gradually, NIHL can remain undetected until symptoms begin to interfere with daily life (13). To identify and quantify hazardous acoustic situations, sound level meters or noise dosimeters provide accurate measurements. However, they are often bulky and expensive devices that are difficult for a layperson to operate. Several studies demonstrated the applicability of smartphones for noise dosimetry using the built-in microphones and dedicated sound level meter apps (12, 14–17). However, the quality of assessment varies widely and depends on the monitoring application, with increasing accuracy seen in newer generations of smartphones and apps (18). Jacobs et al. (12) provide a comprehensive overview of available solutions, which in the case of the National Institute for Occupational Safety and Health (NIOSH) sound level meter app even achieve a measurement accuracy of <2 dBA. Compared to sound level meters or professional noise dosimeters, personal smartphones offer a much more accessible option for monitoring noise environments in everyday life. Yet, two factors limit the feasibility of identifying and quantifying hazardous noise environments with a smartphone. First, people usually keep their smartphones in trouser pockets or handbags, causing sound attenuation due to obstructed microphone openings. Ventura et al. (19) measured an attenuation of 5 dB if the phone was kept inside the pocket. Second, the smartphone always needs to be re-positioned by the user when changing the setting.

In contrast, smartwatches, becoming increasingly common, are worn on a person's wrist and can therefore overcome the limitations associated with smartphones. In this study, we aimed to assess the applicability of using a smartwatch and an integrated noise monitoring application for noise dosimetry. We hypothesized that the position of the watch's microphone on the wrist enables continuous monitoring of acoustic situations to identify and quantify hazardous noise environments. To test the hypothesis, we compared environmental noise measurements taken by a smartwatch with those of a professional sound level meter in everyday scenarios.

## 2. Methods

### 2.1. Study Design

We performed measurements in different recreational and occupational settings in a prospective comparative manner. To maximize representativeness of environmental noise exposure, continuous sound level measurements were performed in 13 different everyday acoustic scenarios. Only the noise levels and no audio files were recorded. According to Swiss legislation, no ethical approval by an institutional review board was required.

### 2.2. Data Collection

**Figure 1:**
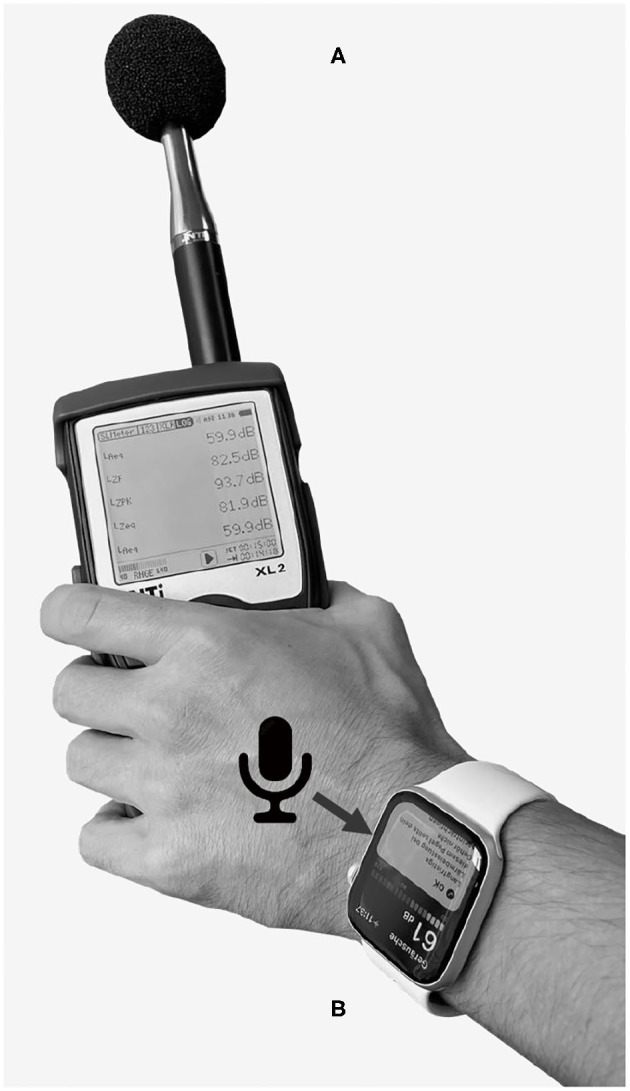
Measurement configuration of the sound level meter **(A)** and smartwatch **(B)** during noise level assessment. The arrow points to the microphone opening of the smartwatch.

A widely adapted metric to quantify noise exposure is the A-weighted equivalent continuous sound pressure level (*L*_*Aeq*_ expressed in dBA). It is defined as the averaged sound pressure level of noise fluctuating over a period of time, weighted by a spectral level correction considering the varying sensitivity of human hearing for different frequencies (20, 21). To measure the *L*_*Aeq*_ levels, we simultaneously used two devices in 13 different acoustic settings. We chose the settings to represent both recreational and workplace noise exposure (see Table 1). For each setting, 12 sample blocks were recorded over a 5 min observation period, resulting in 1 h of recordings per setting and device. We considered the sample block observation time of 5 min long enough to realistically characterize an acoustic environment while providing a sufficiently large sample size. All measurements were recorded by the same investigator with a body height of 175 cm. The first device was a smartwatch (Apple Watch Series 6, Apple Corp., Model A2376, USA) worn on the left wrist of the investigator with the microphone pointing to the back of the hand (Figure 1). The second device was a handheld sound level meter (XL2, NTi Audio, Schaan, Liechtenstein) with a free-field measurement microphone (M2230-WP, NTi Audio) fulfilling class 1 environmental requirements according to IEC 61672. To comply with the recommendations for community measurements, a spectral correction for horizontal sound incidence was activated in the sound level meter. During the measurements, the investigator held the analyzer in his left hand and ensured that the smartwatch's microphone was unobstructed by clothing (Figure 1).

**Table 1:** Overview of settings for environmental noise monitoring.

**Setting**	**Description**
Bar	Conversation of two people sitting at a table positioned next to a wall in a bar. Electronic music played from four loudspeakers placed at the upper corners of a square room, with several other tables occupied.
Canteen	Recorded during a lunch break at a crowded (>100 people) canteen.
Cinema	Recorded during a science-fiction movie in the middle of the cinema hall with a capacity of about 150 people.
Concert	Symphony orchestra and piano playing the Emperor Concerto (Beethoven) and Symphony No. 15 (Shostakovich); seat at the back left of the parquet, all seats occupied.
Construction site	Road construction site including jackhammer noise and construction vehicle noise.
Housekeeping	Recordings during cooking, vacuum cleaning, cleaning out the dishwasher, and dish washing.
Museum	Exhibition of paintings. Walking on different floors (tiles and creaking wooden floorboards). Recordings include announcements over loudspeakers and the collection of the backpack from the cloak room.
Music club	Recorded while dancing on the dance floor at an electronic music event.
Office	Recorded in an office with 4–8 people (30 m^2^). Mainly quiet computer work and short conversations.
Restaurant	Seat at the bar in front of the cooking island, all tables occupied.
Street	Recorded at a busy intersection with urban road traffic.
Commuter train	Seat at the window in a middle row of a full coach. Measurements included announcements over loudspeakers, eating, tunnels, luggage put on rack, conversations, talking, and crying children.
Train station	Recorded at busy main halls of train stations in two central European cities with around 200,000 inhabitants.

On the smartwatch, we measured the environmental noise levels using the integrated “Noise” application (Apple watchOS 8.1). Based on a proprietary algorithm, the application returns levels at adaptively calculated interval lengths ranging from 35 s in quiet situations to 1 s in noisy environments. To enable continuous noise level recording, the smartwatch has to be worn on the wrist with the application actively running. We exported the measurement data *via* the “Health” application running on a paired smartphone (iOS 15.1, iPhone 6s, Apple Corp., USA). Pairing of the smartwatch with the smartphone was performed once for system setup. Subsequently, the smartwatch could be used independently without the need to carry the smartphone around. The handheld sound level meter provided data for 1 s intervals stored with the corresponding time stamps on an integrated memory card. To enable comparison of sample blocks with the same duration and since the smartwatch did not provide samples at regular time intervals like the sound level meter, we used the following equation to calculate the cumulative noise exposure *L*_*Aeq*,Σ_ over a total period *T*, found from *N* individual measurements *L*_*Aeq,i*_ with varying observation lengths *T*_*i*_:


(1) $$\begin{array}{r} L_{Aeq,\Sigma }=10\log_{10}\left(\frac{1}{T}\overset{N}{\underset{i=1}{\sum }}\left(10^{L_{Aeq,i}/10}\cdot T_{i}\right)\right),\text{ with }T=\overset{N}{\underset{i=1}{\sum }}T_{i}. \end{array}$$

### 2.3. Data Analysis

The data were grouped and analyzed separately for occupational settings (*canteen, construction site, office, street, commuter train*, and *train station*) and recreational settings (*bar, cinema, concert, housekeeping, museum, music club*, and *restaurant*), as well as for *non-hazardous* (*L*_*Aeq*_ < 70 dBA), *tolerable* (70 ≤ *L*_*Aeq*_ ≤ 85 dBA), and *hazardous* (*L*_*Aeq*_ > 85 dBA) sound pressure level categories (3).

To evaluate the measurement agreement, we calculated the mean error (ME) and standard deviation (SD) of the measurement differences (i.e., sound level meter minus smartwatch levels) for each setting and for the overall recordings (total of 156 sample block pairs). In addition, a scatter plot with linear regression and a Bland-Altman plot were created (22). The a priori acceptable limits of agreement were chosen with ±2 dBA, following the IEC 61672 and ANSI S1.4 guidelines for class/type 2 sound level meters (12, 23). The normal distribution of the measurement differences was verified by inspecting the histogram. Absolute agreement was also quantified with the intra-class correlation coefficient (ICC). Since each measurement was performed once by both devices by a single investigator, and the devices can be considered representative of a larger population of similar devices, the ICC(2,1) metric was chosen (24). A scatter plot with linear regression and histogram was used to analyze the distribution of sampling intervals of the smartwatch. The statistical analysis was performed using MATLAB (version R2020b; The MathWorks Inc., USA) and the R environment (v4.0.3) (25).

## 3. Results

**Figure 2:**
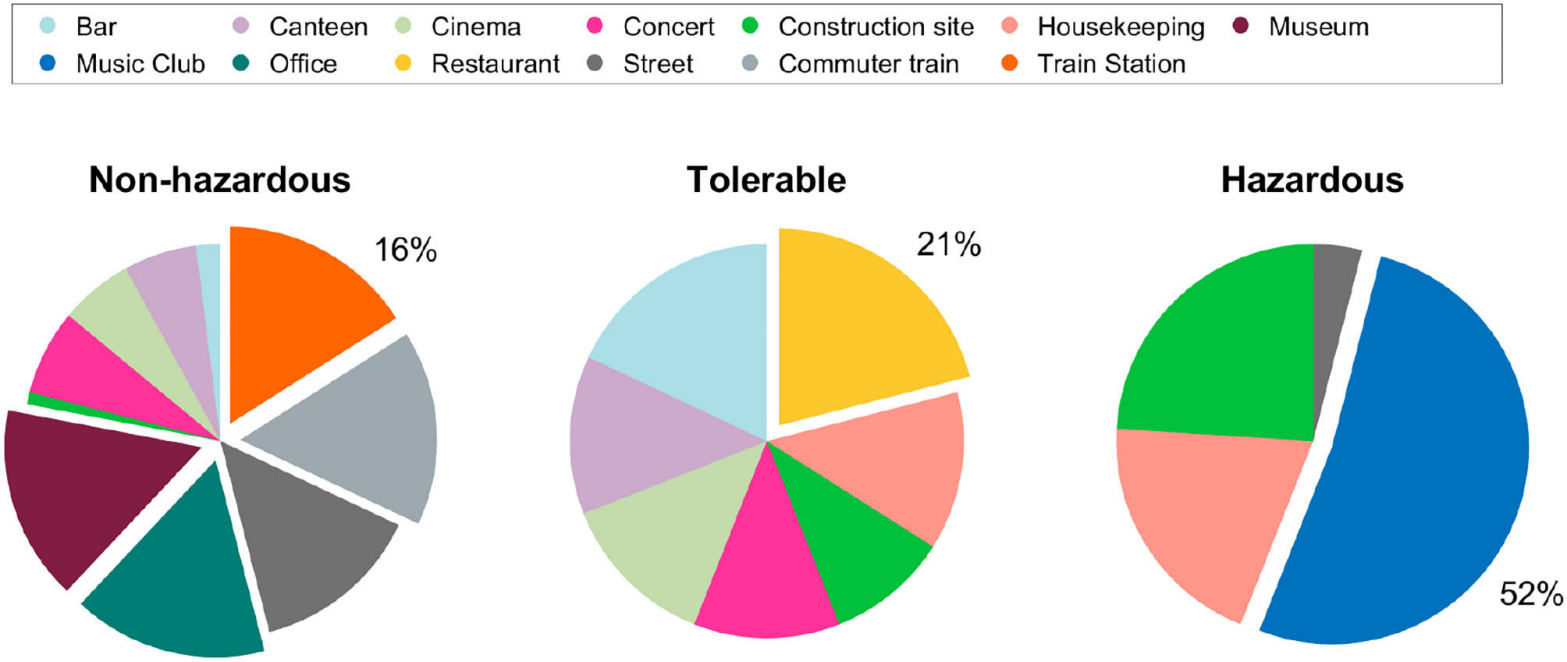
Pie charts of the sample block distribution (mean *L*_*Aeq*_) among the sound level categories.

Table 2 summarizes the noise exposure measurements for all settings and categories. In total, 13 h of environmental noise were monitored with the sound level meter and the smartwatch. Noise exposure was highest in the music club and lowest during office work and the museum visit. The distribution of sample blocks in the settings with *non-hazardous* levels was: *office* (16%), *museum* (16%), *commuter train* (16%), *train station* (16%), *street* (14%), *concert* (7%), *canteen* (6%), *cinema* (6%), *bar* (2%), and *construction site* (1%). Sample blocks with *tolerable* noise levels were distributed as: *restaurant* (21%), *bar* (18%), *canteen* (13%), *cinema* (13%), *housekeeping* (13%), *concert* (12%), and *construction site* (10%). Most of *hazardous* noise levels were measured in the *music club* (52%), followed by the *construction site* (24%), *housekeeping* (20%), and *street* (4%) settings (see Figure 2).

**Table 2:** Summary of noise exposure for the measured settings and categories.

**Setting**	**Sample blocks**	**Noise exposure (dBA)**			

		**Mean** ***L*_*Aeq*_ (Standard deviation)**		**Cumulative** ***L*_*Aeq*, Σ_**	
		**Sound level meter**	**Smartwatch**	**Sound level meter**	**Smartwatch**
Bar	12	71.4 (1.1)	72.1 (2.1)	71.5	72.6
Canteen	12	68.7 (4.7)	69.9 (5.2)	70.5	72.0
Cinema	12	72.2 (4.5)	71.5 (4.2)	74.1	73.2
Concert	12	71.7 (7.1)	71.6 (7.7)	76.4	76.8
Construction site	12	83.2 (6.4)	83.8 (7.7)	86.7	90.0
Housekeeping	12	79.0 (6.6)	80.1 (7.6)	82.3	84.1
Museum	12	55.9 (3.1)	52.9 (5.1)	57.1	55.9
Music club	12	95.7 (1.7)	93.9 (1.8)	96.0	94.3
Office	12	49.4 (5.7)	48.6 (7.5)	52.5	52.2
Restaurant	12	77.5 (1.9)	75.7 (2.2)	77.8	76.2
Street	12	69.4 (5.1)	69.5 (5.8)	75.3	77.4
Commuter train	12	63.5 (3.3)	61.3 (4.4)	64.6	63.2
Train station	12	67.0 (1.8)	66.6 (1.6)	67.3	66.9
**Category**					
Occupational settings	72	66.9 (11.0)	66.6 (12.0)	79.4	82.5
Recreational settings	84	74.8 (11.8)	74.0 (12.4)	87.9	86.4
Non-hazardous levels	77	61.9 (7.5)	61.0 (8.4)	65.5	65.3
Tolerable levels	56	75.6 (4.3)	75.1 (3.6)	77.8	76.9
Hazardous levels	23	92.1 (4.8)	90.8 (4.2)	94.1	92.7
**Overall**	156	71.1 (12.1)	70.6 (12.7)	85.7	85.0

**Figure 3:**
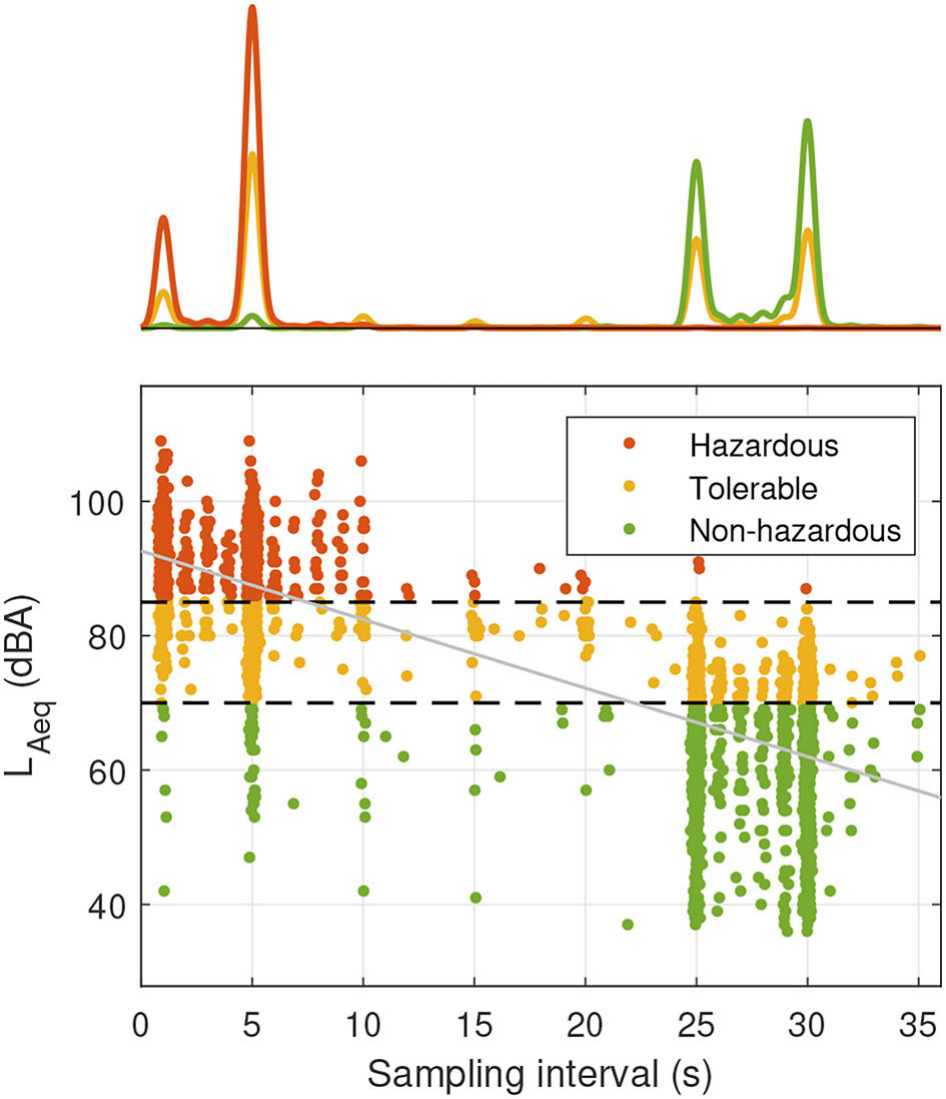
Scatter plot with histogram representing the relationship between smartwatch samples *L*_*Aeq*_ and sampling interval for different sound pressure level categories. The smartwatch sampling intervals are automatically adjusted by the device and have integer values of seconds; jitter was added to improve visibility. The black dashed lines indicate the threshold values for sound pressure level categorization (70 and 85 dBA, respectively). The least-squares regression line is depicted in gray.

The association between the *L*_*Aeq*_ and the smartwatch's sampling interval is depicted in Figure 3. The higher the *L*_*Aeq*_ smartwatch samples, the lower the corresponding sampling interval (i.e., the more samples were measured during the observation period) and vice versa. Most of the *L*_*Aeq*_ smartwatch samples in the *hazardous* category were measured with a sampling interval ≤10 s, whereas most of *L*_*Aeq*_ smartwatch samples in the *non-hazardous* category were measured with a sampling interval ≥25 s. The regression analysis confirmed the significant linear relationship (slope = −1.02; 95% CI = [−1.04, −1.00]; *r*^2^ = 0.65).

Overall, the ME of the *L*_*Aeq*_ differences between the sound level meter and the smartwatch was 0.5 dBA (SD of 1.8 dBA) indicating a slight underestimation by the smartwatch (see Table 3). Because the 95% confidence interval (CI) of the ME does not contain zero, the averaged measurement offset between the sound level meter and the smartwatch can be considered statistically significant (26). Non-significant measurement bias was observed for the settings *concert, construction site, office*, and *street*. In the grouped analysis, a bias was observed for *recreational* settings and *non-hazardous* sound pressure levels, where the smartwatch underestimated the *L*_*Aeq*_ levels.

**Table 3:** Noise exposure differences and intra-class correlation coefficient (ICC) between sound level meter and smartwatch.

**Setting**	***L***_***Aeq***_ **difference (dBA)**			**ICC**

	**ME (SD)**	**95% CI**	**95% LOA**	
Bar	−0.8 (1.1)	[−1.5, −0.1]	[−3.0, 1.5]	0.73
Canteen	−1.2 (1.0)	[−1.8, −0.6]	[−3.1, 0.7]	0.95
Cinema	0.7 (0.7)	[0.3, 1.2]	[−0.7, 2.1]	0.97
Concert	0.1 (0.8)	[−0.4, 0.6]	[−1.5, 1.6]	0.99
Construction site	−0.5 (1.9)	[−1.7, 0.7]	[−4.1, 3.1]	0.97
Housekeeping	−1.1 (1.3)	[−1.9, −0.3]	[−3.5, 1.4]	0.98
Museum	2.9 (2.2)	[1.5, 4.3]	[−1.3, 7.2]	0.71
Music club	1.8 (1.2)	[1.0, 2.5]	[−0.6, 4.1]	0.65
Office	0.8 (2.2)	[−0.6, 2.2]	[−3.5, 5.2]	0.94
Restaurant	1.8 (0.6)	[1.4, 2.1]	[0.6, 2.9]	0.71
Street	−0.1 (1.5)	[−1.1, 0.8]	[−3.0, 2.8]	0.97
Commuter train	2.2 (1.3)	[1.4, 3.0]	[−0.3, 4.8]	0.82
Train station	0.3 (0.4)	[0.1, 0.6]	[−0.5, 1.1]	0.96
**Category**				
Occupational settings	0.3 (1.8)	[−0.2, 0.7]	[−3.3, 3.8]	0.99
Recreational settings	0.8 (1.8)	[0.4, 1.2]	[−2.7, 4.4]	0.99
Non-hazardous levels	1.1 (1.8)	[0.7, 1.5]	[−2.5, 4.6]	0.97
Tolerable levels	0.1 (1.5)	[−0.3, 0.5]	[−2.7, 2.9]	0.96
Hazardous levels	0.3 (2.0)	[−0.6, 1.2]	[−3.6, 4.3]	0.90
**Overall**	0.5 (1.8)	[0.2, 0.8]	[−3.0, 4.0]	0.99

*ME, mean error; SD, standard deviation; CI, confidence interval; LOA, limits of agreement*.

Comparison of all 156 *L*_*Aeq*_ values showed excellent agreement between the measurement devices (i.e., ICC>0.9) (27). The lowest ICC scores were found for the measurements performed in the *music club*, the *restaurant*, and the *museum* settings (ICC = 0.65, 0.71, and 0.71, respectively). The best ICC scores were observed for the *concert* and the *housekeeping* settings (ICC>0.99, ICC = 0.98, respectively). Excellent absolute agreement was found independent of the noise level category.

**Figure 4:**
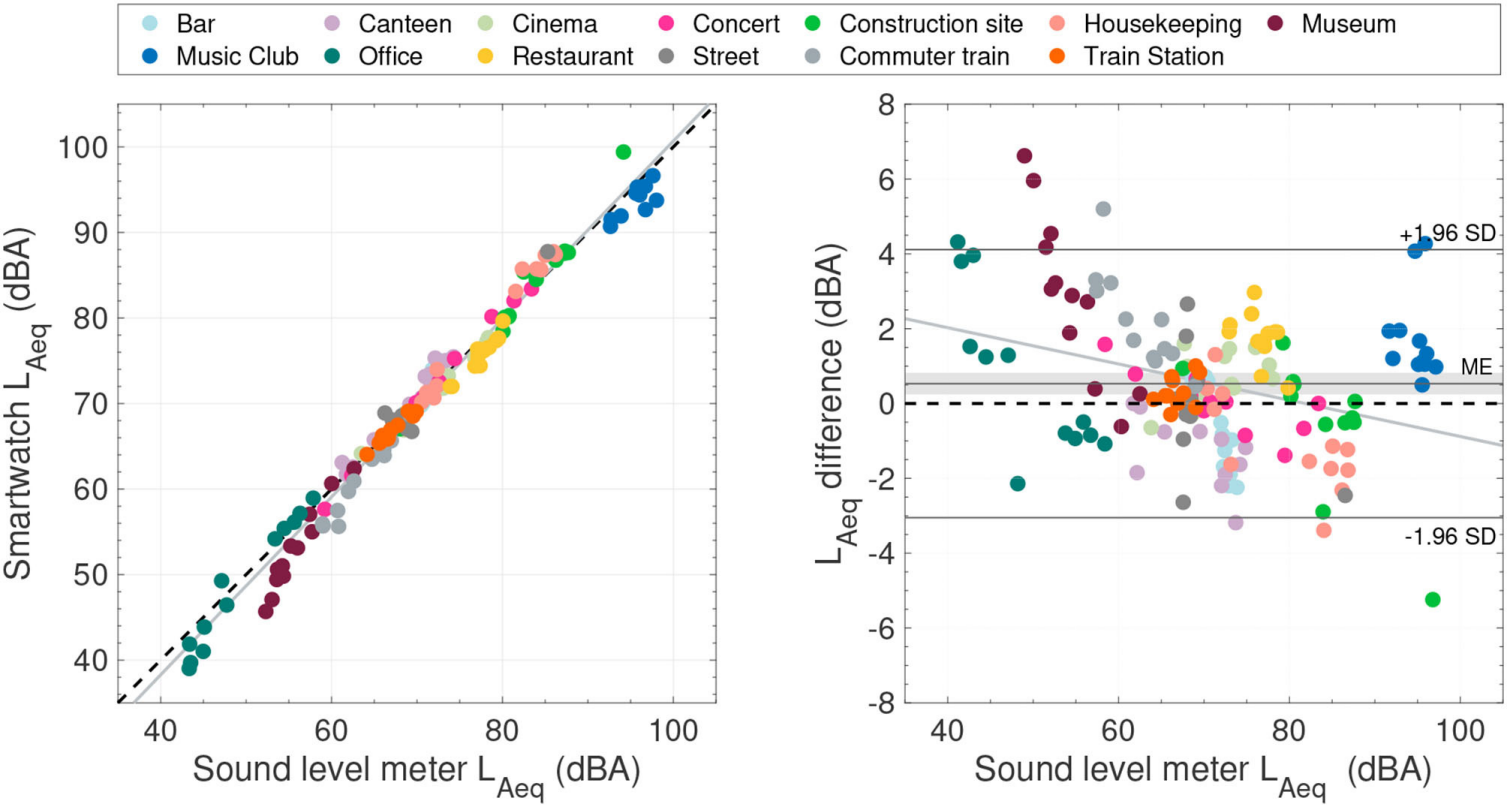
**(Left)** Scatter plot comparing the *L*_*Aeq*_ sample blocks between the sound level meter and the smartwatch. The black dashed line indicates identical measurements. **(Right)** Bland-Altman plot using the sound level meter as reference. The gray-shaded area indicates the 95% confidence interval of the mean error (ME). In both plots, the gray lines represent the least-squares regression line. The measurement settings are color encoded.

Figure 4 visualizes the data in a scatter plot and a Bland-Altman plot. As expected, when comparing two methods designed to measure the same variable, the regression analysis showed a high correlation between the two devices (slope = 1.04; 95% CI = [1.02, 1.06], *r*^2^ = 0.98). The Bland-Altman plot illustrates the small but statistically significant measurement bias of 0.5 dBA (95% CI = [0.2, 0.8]). Overall, the 95% limits of agreement ([−3.0, 4.0], see Table 3) exceed the a priori accepted limits of ±2.0 dBA. The slope of the regression line in the Bland-Altman plot (−0.04; 95% CI = [−0.06, −0.02], *r*^2^ = 0.07) indicates a proportional bias between the two devices. The a priori limits of agreement were met for the *concert* and *train station* settings, and the limits were exceeded for all other settings or categories.

## 4. Discussion

In this study, we examined the applicability of a popular smartwatch for environmental noise monitoring in various occupational and recreational settings. Ultimately, we questioned if their noise measurement capabilities could improve public health awareness. Overall, the ICC showed excellent agreement [ICC>0.9, (27)] between the smartwatch and the reference sound level meter. A small but statistically significant measurement bias of 0.5 dBA toward underestimation by the smartwatch was found. Moreover, the limits of agreement exceeded the a priori defined acceptable limits of agreement for high-accuracy noise exposure assessment. The mean *L*_*Aeq*_ value of the sound level meter for the *restaurant* was in line with environmental noise measurements performed in Jacobs et al. (12) [measured with a calibrated Edge eg5 noise dosimeter (3 M, St. Paul, USA)]. For the *commuter train* and the *office* we found 10.1 and 4.3 dBA lower mean *L*_*Aeq*_ values as in Jacobs et al. (12), respectively. The *music club* recordings in this study had 2.2 dBA lower mean *L*_*Aeq*_ values as in Williams et al. (28) [measured with a calibrated CEL-350 dBadge noise dosimeter (Caselle-CEL, Bedford, UK)]. The *cinema* mean *L*_*Aeq*_ value was slightly lower than the range given in Ferguson et al. (29), who measured with a sound level meter (Bruel and Kjaer, type 2260) and a noise dosimeter (Quest, Q-400). Mean *L*_*Aeq*_ *street* noise was 3 dBA lower compared to the measurements of McAlexander et al. (30) (Q-300 noise dosimeter, Quest, Oconomowoc, USA). Also for the *concert*, the *bar*, the *music club* and the *restaurant*, lower values as in Beach et al. (31) were recorded (measured with a CEL-350 dBadge noise dosimeter).

Measurement agreement between the devices varied depending on the acoustic environment, with limits of agreement ranging over 2.7 dBA (*restaurant*) to 8.7 dBA (*office*). Our recordings may have been influenced by environmental (cultural setting, measurement timing, room size, and occupation type) and device-related (microphone directionality and algorithmic implementation) factors. Jacobs et al. (12) assessed the accuracy of the NIOSH smartphone noise dosimeter app and observed that settings with rapid acoustic fluctuations tended to be measured less accurately than settings with slower acoustic fluctuations. In contrast to Jacobs et al. (12), we assumed that the microphone orientation of the two devices was not responsible for this discrepancy because the microphone/smartwatch arrangement was independent of the acoustic environment. Rather, we hypothesized a relationship in the number of *L*_*Aeq*_ samples per time interval and the dynamics of the acoustic environment (especially self-movement and movement of sound sources). On average, the smartwatch's noise estimations were based on fewer *L*_*Aeq*_ samples compared to the sound level meter samples available. The contrast between the 1 s sampling interval of the sound level meter to the sampling intervals of the smartwatch is depicted in Figure 3. This observation led us to the assumption that a lower sampling rate of the smartwatch could have influenced measurement accuracy. Compared to the *hazardous* environments, only *non-hazardous* settings revealed a significant *L*_*Aeq*_ difference between the smartwatch and the sound level meter (see Table 3). However, despite the decrease in the number of smartwatch *L*_*Aeq*_ samples available in the *non-hazardous* environments (Figure 3), the SD of *L*_*Aeq*_ differences and the ICC between smartwatch and sound level meter was comparable across sound pressure levels (SD: 1.8, 1.5, and 2.0; ICC: 0.97, 0.96, and 0.90 for the *non-hazardous, tolerable*, and *hazardous* categories, respectively; Table 3). Hence, we concluded that a low sampling rate of the smartwatch did not necessarily result in a poor agreement between the devices. Future work may quantitatively investigate the hypothesis that the measurement accuracy of the smartwatch was directly dependent on the dynamics of the acoustic environment. To capture the influence of hand and wrist motion, data from the smartwatch's inertial measurement unit could be recorded during environmental noise measurements in simulated or real dynamic acoustic environments (32, 33).

While occupational noise exposure is known to cause NIHL, recently experts have highlighted the dangers posed by exposure to loud noises outside the workplace (3, 12, 34). Both *recreational* and *occupational* settings were evaluated in our study. Besides a small but statistically significant measurement bias, the measurement agreement between the sound level meter and the smartwatch was considered similar for both settings. This suggests that the smartwatch is equally well applicable in *recreational* and *occupational* settings. Furthermore, the excellent overall measurement agreement between the smartwatch and the reference sound level meter confirmed our primary hypothesis. Smartwatches provide an easy-to-use but also accurate way to identify and quantify hazardous noise environments in everyday life. For example, we found a significant amount of hazardous sound pressure levels at home during housekeeping, an environment that could generally be thought to be less affected by noise. Therefore, we believe smartwatches can play an important role as a means of hearing protection by raising awareness of personal noise exposure. In the future, we can envision the clinical application of smartwatches for audiological or neuro-otological diagnostics and follow-up purposes. Patients with inner ear disorders, including hearing loss, tinnitus, or vertigo, could be provided with a smartwatch to continuously monitor diagnostic markers, including noise exposure, gait, and other vital parameters. In addition, noise measurements of smart devices can be used as control parameter for hearing aids and implant audio processors, e.g., to support noise suppression algorithms (35–37). For legally relevant environmental noise assessments that require high accuracy, we recommend professional sound level meters or noise dosimeters.

## 5. Conclusions

Smartwatches provide a user-friendly, easily accessible and discreet alternative for reliable continuous noise monitoring in recreational and occupational environments. Because of small measurement inaccuracies, legally binding or high-accuracy noise assessment should be conducted using professional sound level meters or noise dosimeters. However, as people increasingly use smartwatches in their daily lives, they are a powerful tool to raise the awareness to individual hearing protection. Moreover, we believe that smartwatch-based noise exposure monitoring, and health parameter monitoring in general, will be clinically relevant in the future to prevent NIHL and tinnitus.

## Data Availability Statement

The raw data supporting the conclusions of this article will be made available by the authors, without undue reservation.

## Author Contributions

TF: methodology, software, formal analysis, visualization, and writing—original draft. SS: investigation, data curation, and writing—review and editing. MC: supervision, project administration, and funding acquisition. WW: conceptualization, methodology, formal analysis, writing—review and editing, project administration, and funding acquisition. All authors contributed to the article and approved the submitted version.

## Funding

This work was in part supported by the Alois und Irma Weber-Goldinger-Stiftung, the Dr. Jean Stieger-Stiftung, and the Uranus foundations. Open access funding provided by University of Bern.

## Conflict of Interest

The authors declare that the research was conducted in the absence of any commercial or financial relationships that could be construed as a potential conflict of interest.

## Publisher's Note

All claims expressed in this article are solely those of the authors and do not necessarily represent those of their affiliated organizations, or those of the publisher, the editors and the reviewers. Any product that may be evaluated in this article, or claim that may be made by its manufacturer, is not guaranteed or endorsed by the publisher.
